# A Systematic Review: Does Insulin Resistance Affect the Risk and Survival Outcome of Breast Cancer in Women?

**DOI:** 10.7759/cureus.21712

**Published:** 2022-01-29

**Authors:** Mirra Srinivasan, Hadia Arzoun, Lekshmana Bharathi GK, Santhosh Raja Thangaraj

**Affiliations:** 1 Internal Medicine, California Institute of Behavioral Neurosciences & Psychology, Fairfield, USA; 2 Internal Medicine, Rajah Muthiah Medical College and Hospital, Chidambaram, IND

**Keywords:** outcomes, cohort study, association, metabolic syndrome, risk, breast cancer, insulin resistance

## Abstract

Currently, breast cancer is one of the insidious malignancies that evolves silently, eventually leading to mortality, and has been recorded as one of the leading causes of cancer-related deaths around the globe. It is evident from numerous research studies that the etiology of breast cancer is multifaceted, both on an individual and environmental level. Insulin resistance, commonly known as metabolic syndrome, has been related to a poor breast cancer prognosis. There is presently limited data on the clinical features of insulin-resistant breast cancer patients. The purpose of this study is to examine the association between metabolic syndrome and its components impacting the risk and the prognosis of breast cancer, including the clinicopathological variables in patients with newly diagnosed breast cancer with and without already established diabetes. The authors extracted data from PubMed, Google Scholar, Science Direct, and Embase, intending to study the connections between these two entities. Studies from worldwide were selected to analyze the association between breast cancer and insulin resistance, including mammogram screening practices in a region-wise manner. The ultimate objective is to raise awareness of this association among practitioners worldwide. After analyzing several reports that included observational studies, it is to be concluded that insulin resistance impacts breast cancer both in regards to increasing the risk as well as affecting the survival outcome.

## Introduction and background

Breast cancer (BC) is the most prevalent cancer in women, accounting for more than one out of 10 new cancer diagnoses every year, and is the second leading cause of cancer-related mortality among women worldwide [1]. BC affects women of all ages after puberty and the incidence increases as they become older [2]. According to the National Breast Cancer Coalition, a woman dies from BC every 13 minutes, and approximately four million people in the United States are living with a history of BC as of 2019 [3]. BC has been diagnosed in 7.8 million women in the previous five years, thus leading to more disability-adjusted life years (DALYs) in women worldwide than any other kind of cancer. From the 1930s until the 1970s, BC mortality did not alter much, and it was not until the 1980s that the survival rates began to improve in nations with early diagnosis programs and novel treatment options to combat invasive cancer [2].

BC impacts all elements of a woman's life, including physical, emotional, and social well-being. Although the specific etiology of BC is unknown, risk factors have been recognized [4], such as female gender [2], aging, a family history of BC, particular abnormalities in the breast(s) [4], mutations in the breast cancer gene 1 (BRCA1), breast cancer gene 2 (BRCA2), and partner and localizer of BRCA2 (PALB-2), reproductive history (such as the age at which menstrual periods began and the age at which the first pregnancy occurred), cigarette use, postmenopausal hormone treatment factors [2], lack of physical activity, alcohol intake, obesity, diet, race, and chest radiation therapy [4]. Unfortunately, even if all of the theoretically modifiable risk factors were addressed, the probability of getting BC would be reduced by no more than 30% [2]. Based on the presence or absence of molecular markers for estrogen or progesterone receptors and human epidermal growth factor 2 (ERBB2; formerly HER2), BC is divided into three subtypes: hormone receptor-positive/ERBB2 negative (70% of patients), ERBB2 positive (15-20%), and triple-negative (tumors lacking all three standard molecular markers; 15%) [5].

Insulin resistance (IR), generally an acquired disorder, is defined as a reduced physiologic response to insulin stimulation in target tissues [6] such as the liver, muscle, and adipose tissue and thus hinders glucose disposal, leading to an increase in beta-cell insulin synthesis and hyperinsulinemia as a compensatory response [7]. IR has been linked to increased body fat, while hereditary factors have also been discovered, and since there is no universally acknowledged test for IR, the clinical definition remains ambiguous [7,8]. IR is diagnosed clinically by metabolic alterations such as hyperglycemia, hypertension, dyslipidemia, visceral obesity, hyperuricemia, increased inflammatory markers, endothelial dysfunction, and a prothrombic state [7].

"The insulin resistance syndrome" or "the metabolic syndrome (MetS)" is viewed as a group of cardiovascular-metabolic disorders where multiple hypotheses have been postulated, such as genetic anomalies of one or more proteins in the insulin action cascade, fetal malnutrition, and increased visceral adiposity [9]. The MetS is a collection of illnesses that, when combined, increase the risk of atherosclerotic cardiovascular disease, IR, diabetes mellitus, and vascular and neurological consequences such as a cerebrovascular accident [10]. The presence of three out of five of the following components was categorized as MetS: waist circumference > 88 cm, blood pressure > 130/85 mmHg, serum triglycerides > 150 mg/dL, high-density lipoprotein > 50 mg/dL, and fasting glucose > 110 mg/dL [11].

Previous research studies have looked into the association between MetS and cancer, including breast, colon, and endometrial malignancies, for which MetS was considered a risk factor [6]. It is also to be noted that women with IR are more likely to acquire proliferative malignancies, especially BC, and may even have a poorer prognosis. Although the mechanisms behind these links are unknown, multiple investigations have indicated that IR induces chronic persistent hyperinsulinemia, which is thought to play a role in carcinogenesis [6].

This review mainly focuses on connecting the dots between IR and the increased risk and survival outcomes of BC, with the sole purpose of recognizing these individuals and ultimately correcting the metabolic derangements, which may prevent incidence or even decrease the recurrence rate of BC in women, thus avoiding the evolution of malignancy.

Methodology

**Figure 1:**
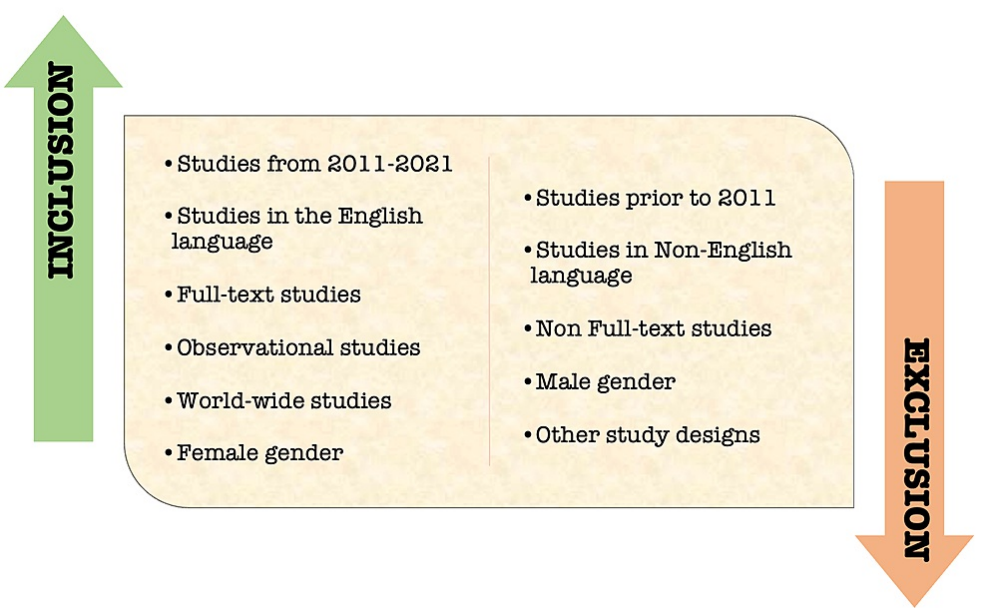
Inclusion and exclusion criteria of the selected studies

A literature search was done in PubMed, Google Scholar, Embase, and Science Direct, with regular and Medical Subject Heading (MeSH) keywords listed below using the Boolean scheme. A total of 1,152 articles were found, and 104 duplicates were removed. A total of 1,048 reports were sought for screening; 932 were excluded based on the titles and abstracts, 33 reports were excluded due to irrelevancy, and 75 reports were excluded based on the exclusion criteria. Finally, eight reports were included in this review as eligible and final studies. The inclusion and exclusion criteria set for this review are depicted in Figure 1.

**Figure 2:**
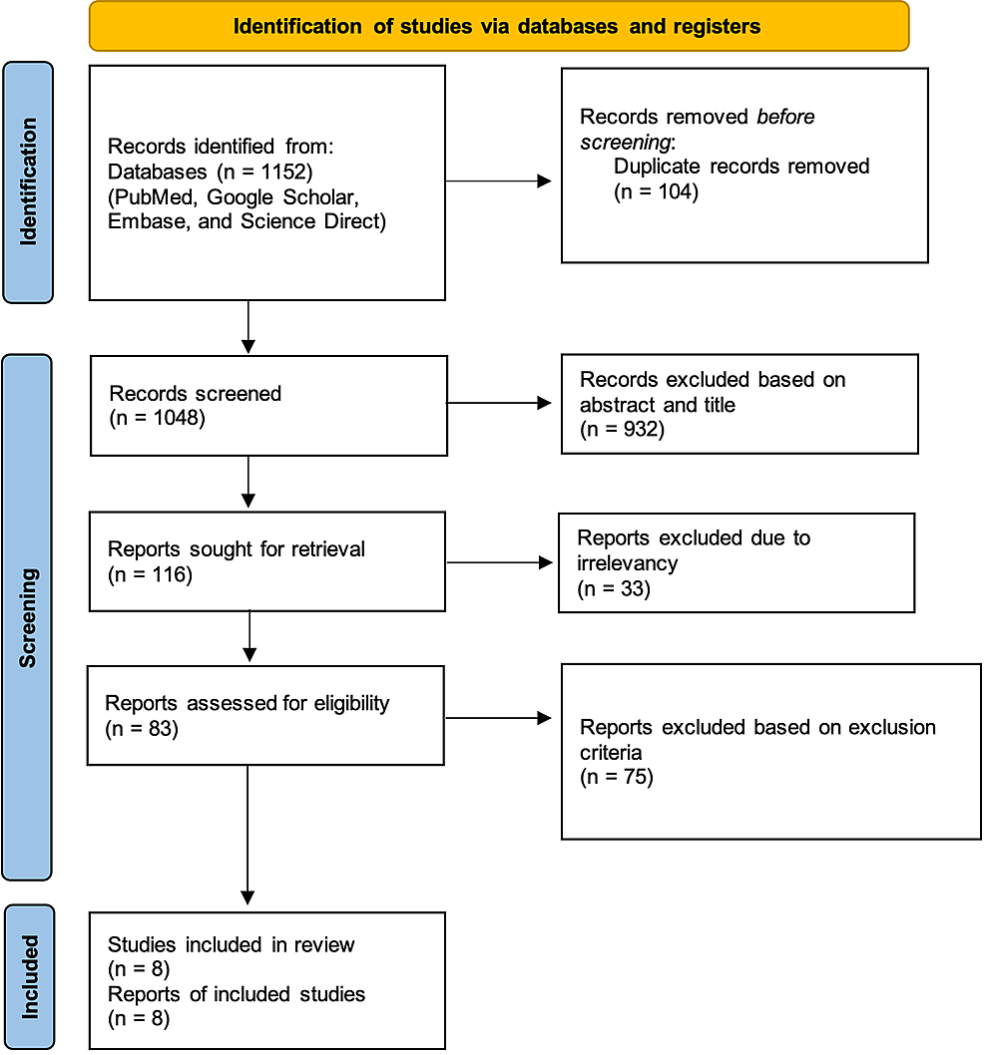
PRISMA flow chart PRISMA: Preferred Reporting Items for Systematic Reviews and Meta-Analyses.

This systematic study followed the Preferred Reporting Items for Systematic Reviews and Meta-Analyses (PRISMA) 2020 guidelines and principles. Figure 2 below illustrates the PRISMA flow chart [12].

Keywords

MeSH Keywords

Breast cancer risk OR (("Breast Neoplasms/diagnosis"[Mesh] OR "Breast Neoplasms/etiology"[Mesh] OR "Breast Neoplasms/pathology"[Mesh] OR "Breast Neoplasms/prevention and control"[Mesh] )) AND insulin resistance OR metabolic syndrome OR ("Insulin Resistance/complications"[Mesh] OR "Insulin Resistance/metabolism"[Mesh] OR "Insulin Resistance/pathology"[Mesh]).

Keywords in Other Databases

Keywords searched in other databases include insulin resistance, breast cancer, risk, metabolic syndrome, association, cohort study, and outcomes.

Quality assessment

The authors independently assessed the quality of the included studies using appropriate quality appraisal methods; in the event of a disagreement among the authors, a third author was consulted, and disputes were addressed to establish a common ground. The examined studies were awarded appropriate scores, as shown in Table 1.

**Table 1:** Quality appraisal for the included studies

Type of study	Number of studies	Quality appraisal tool	Scores
Prospective cohort	6	Newcastle-Ottawa Scale	≥10
Retrospective cohort	1	Newcastle-Ottawa Scale	≥10
Case-control	1	Newcastle-Ottawa Scale	≥10

Results

The individual characteristics of the included studies are summarized in Table 2.

**Table 2:** Characteristics of the included studies MetS: metabolic syndrome.

Author	Year	Study design	Region	Subjects	Study about the risk or survival outcome
Duggan et al. [13]	2011	Prospective cohort	United States (New Mexico, Seattle, Los Angeles)	527	Survival outcome
Sieri et al. [14]	2012	Prospective cohort	Italy (Lombardy)	10,786	Risk
Zhang et al. [15]	2012	Retrospective cohort	China (Jiaxing city)	7,950	Risk
Jiralerspong et al. [16]	2013	Prospective cohort	United States (Texas)	6,342	Survival outcome
Nam et al. [6]	2016	Prospective cohort	Korea (Seoul)	1,301	Survival outcome
Kabat et al. [17]	2017	Prospective cohort	United States (40 clinical sites across the nation)	21,000	Risk
Shu et al. [18]	2018	Case-control	European descent	Cases: 98,842; controls: 83,464	Risk
Buono et al. [11]	2020	Prospective cohort	Italy (Pascale, Naples)	MetS = 173; non-MetS = 544	Survival outcome

Table 3 provides a detailed analysis of the relevant findings and the conclusions of the included studies.

**Table 3:** Key findings and conclusions of the included studies HOMA: homeostatic model assessment; BC: breast cancer; RR: relative risk; HOMA-IR: homeostasis model assessment-insulin resistance; SHBG: sex hormone-binding globulin; IR: insulin resistance; T2DM: type 2 diabetes mellitus; SIR: standardized incidence ratio; HRs: hazard ratios; RFS: recurrence-free survival; OS: overall survival; BCSS: breast cancer-specific survival; BMI: body mass index; ER: estrogen receptor; HER2: human epidermal growth factor receptor 2; HR: hazard ratio; MetS: metabolic syndrome; OR: odds ratio; SD: standard deviation; WHR: waist-hip-ratio; DFS: disease-free survival.

Author	Findings	Conclusion
Duggan et al. [13]	The HOMA score was used to assess the relationship between adiponectin, insulin, and glucose levels. Increasing HOMA scores were linked to a lower BC survival rate and a lower overall survival rate. Adiponectin levels were linked to a prolonged survival time in women with BC.	High HOMA scores and low adiponectin levels, both of which are linked to obesity and increased BC mortality.
Sieri et al. [14]	Women in the highest glucose quartile had a substantially higher risk of BC (RR = 1.63; P = 0.003) than those in the lowest glucose quartile. The correlation was substantial in premenopausal and postmenopausal women, and women diagnosed beyond 55 years. Women in the highest quartile of the HOMA-IR had a greater risk of BC than those in the lowest quartile (RR = 1.44). In women diagnosed after 55 years, there was a substantial increase in BC risk in the highest HOMA-IR quartile; in the same group, lower BC risk was significantly related to high SHBG.	Hyperglycemia and IR both increase the possibility of developing BC.
Zhang et al. [15]	Men with T2DM had a cancer rate of 1083.6 per 10^5^ individuals, whereas women with T2DM had a rate of 870.2 per 10^5^. In addition to pancreatic, kidney, liver cancers, and leukemia, an increased risk of developing BC was seen primarily in T2DM women, where the SIR was 2.209.	Female patients with T2DM have a higher chance of developing BC.
Jiralerspong et al. [16]	The HRs for RFS, OS, and BCSS for overweight were 1.18, 1.20, and 1.21, respectively, in a multivariate model adjusted for BMI, diabetes, medical comorbidities, patient- and tumor-related factors, and adjuvant therapy relative to the normal weight. The obese had HRs of 1.13, 1.24, and 1.23 for RFS, OS, and BCSS, respectively. According to subset analyses, these differences were significant for the ER-positive group but not for the ER-negative or HER2-positive groups. Compared to nondiabetics, the HRs for RFS, OS, and BCSS for diabetes were 1.21, 1.39, and 1.04, respectively.	Controlling obesity and diabetes might help BC patients live longer.
Nam et al. [6]	IR was found in 26.4%, and upon multivariate analysis, it was substantially related to older age, obesity, larger tumor size, advanced stage, and high proliferative luminal B/HER2-negative subtype in postmenopausal women. In premenopausal women, however, only obesity was associated with IR.	IR may play a role in tumor growth and may be a useful prognostic indicator as a therapeutic target in postmenopausal BC patients.
Kabat et al. [17]	Obesity was known to increase the risk of BC, irrespective of metabolic health. Obesity and metabolic disease were associated with a significant risk, where the HR was 1.62. Women who had never received hormone treatment exhibited a more vital link to this association.	High levels of HOMA-IR, in addition to obesity, may be an independent risk factor for BC. Insulin sensitivity had a more vital link to risk than the MetS or its components.
Shu et al. [18]	Fasting insulin (OR = 1.71 per SD increase, P = 5.09 × 10^–4^), two-hour glucose (OR = 1.80 per SD increase, P = 4.02×10^–4^), BMI (OR = 0.70 per five-unit rise, P = 5.05×10^–19^, and WHR adjusted BMI (OR=0.85, P=9.22×10^-6^) all had associations with BC risk. Stratified analyses revealed that genetically predicted fasting insulin was more closely associated with the risk of ER-positive cancer. In contrast, associations with two-hour glucose, BMI, and WHR adjusted BMI was consistent regardless of age, menopausal status, ER status, or family history of BC.	Obesity and glucose/insulin-related characteristics that are genetically determined significantly influence the etiology of BC.
Buono et al. [11]	Compared to non-MetS patients, MetS patients had a numerically greater chance of recurrence (DFS: P = 0.07), as well as a considerably increased risk of mortality (OS: P < 0.0001; BCSS: P = 0.001). Furthermore, individuals with one to two MetS components had a higher risk of death than those with 0 components (OS: P = 0.01; BCSS: P = 0.02).	MetS attributed to a poor prognosis in early-stage BC patients. In individuals who do not meet all of the MetS diagnostic criteria, the presence of one or two of the syndrome's components may indicate a poor prognosis.

## Review

The following provides an analysis of the relationship shared by IR and BC regarding the increased risk of developing BC and the variations seen in survival outcomes of already diagnosed BC. The limitations of this review article are also acknowledged at the end of this section.

Insulin resistance and risk of breast cancer

The positive correlation of homeostasis model assessment-insulin resistance (HOMA-IR) with BC risk in an Italian nested case-control study strongly implies that IR plays a role in BC pathogenesis and that MetS is directly and significantly linked to postmenopausal BC risk. Sex hormone-binding globulin (SHBG) levels that are inversely associated with BC risk also suggest an IR role in BC development beyond age 55 since high SHBG levels are associated with enhanced insulin sensitivity, and low SHBG levels are associated with the development of type 2 diabetes mellitus (T2DM) in both men and women. This type of IR may increase the bioavailability of estradiol and testosterone, eventually stimulating testosterone production in the ovaries and adrenal glands, as well as aromatase and 17ß hydroxysteroid dehydrogenase production in adipose tissue, and ultimately producing estradiol. The abundance of these sex hormones accelerates mammary gland epithelial cell growth while inhibiting apoptosis, thus enabling damaged, precancerous cells to escape destruction [14].

In the Chinese population with a considerably leaner and lower body mass index (BMI) (study subjects had a mean BMI of 23.6), the crude incidence rate of BC in females with T2DM was about 182.1. Several mechanisms have been hypothesized to explain the relation between T2DM and cancer risk, especially as both share several risk factors, such as age, obesity, greater saturated fat and refined carbohydrate consumption, sedentary lifestyle, cigarette use, and some psychological factors. T2DM, on the other hand, arises as a result of relative insulin insufficiency and is characterized by compensatory hyperinsulinemia and IR. Insulin is a well-known growth factor that increases cancer cell proliferation and, as a result, may have a role in carcinogenesis. In summary, diabetes was related to higher mortality compared to normoglycemic persons across all cancer types, according to a meta-analysis of 23 studies comprising pre-existing diabetes mellitus [15].

In obesity and T2DM, adipose tissue inflammation is common, and proinflammatory cytokines and prostaglandin E2 generated by cyclooxygenase-2 in invading macrophages cause enhanced aromatase expression. In animal models, the same proinflammatory mediators, as well as the chemokine monocyte chemoattractant protein-1, increase tumor-related angiogenesis by directly stimulating tumor cell proliferation and invasion. This accompanying inflammation increases estrogen-independent, particularly triple-negative, BC growth, invasion, and metastasis through processes involving macrophage-secreted cytokines, adipokines, and insulin, especially before menopause [19].

When overweight and obese patients are combined, the trend toward increased risk with increasing BMI along with the metabolic state is diluted. The data in Kabat et al.'s study implied that increased adiposity (as defined by BMI or waist circumference) and metabolic state both contribute to the risk of BC in separate and combined ways. The existence of the MetS and its components (other than waist circumference) were shown to have modest or nonsignificant relationships with BC. On the other hand, HOMA-IR was shown to be significantly associated with risk, and the MetS' link to BC appears to mirror this since HOMA-IR is strongly linked to the MetS. Obesity and MetS share molecular mechanisms that influence the risk of postmenopausal BC. IR can be a result of obesity, but it can also be influenced by hereditary factors. Increased estrogen levels caused by the aromatization of androgens in adipose tissue may increase breast tissue cell growth. Secondly, increased insulin concentrations caused by IR may have pro-mitotic and anti-apoptotic effects in BC cells, as well as drive cell-cycle progression. Long-term hyperinsulinemia may also result in higher levels of free or bioactive insulin-like growth factor 1 (IGF-1), which can enhance tumor-promoting signaling pathways [17].

The findings of Shu et al.'s study give significant evidence for a favorable relationship. Insulin is a crucial growth agent that promotes cancer by boosting cell mitosis and migration while also suppressing apoptosis. Its mitogenic effects are mediated via the activation of Ras and the mitogen-activated protein kinase (MAPK) pathway, both of which have been linked to cancer formation. Insulin and IGF-1 receptor knockdown have also been found to suppress hormone-dependent proliferation of estrogen receptor (ER)-ß BC cells. This might explain why fasting insulin was linked to ER-ß BC in this research [18].

Insulin resistance and survival outcome of breast cancer

One other study observed a stronger association of mortality with homeostatic model assessment (HOMA) in women (BMI > 25 kg/m2) than insulin concentrations because a single measure of insulin could be an imprecise predictor of long-term insulin production and may not capture the diurnal fluctuation. In addition, HOMA was linked to all-cause and BC mortality in patients diagnosed with regionally invasive and ER-positive BC. Insulin can stimulate cell proliferation in normal and malignant breast cell lines, perhaps through signaling through its receptor. Insulin also inhibits IGF-1-binding proteins and SHBG, in turn increasing the bioavailable mitogens. An earlier report from the same study found that elevated C-peptide concentrations were negatively associated with BC survival [13].

In contrast, the study revealed that adiponectin levels are negatively related to sex steroid levels, which have been linked to BC development. Adiponectin is a hormone primarily produced by adipocytes and may regulate energy intake and expenditure. It influences cell proliferation and cytokine production. In vitro and animal studies show that adiponectin and ERs interact, thus explaining the connection between adiponectin and BC mortality, which is less after adjusting for central obesity (waist-to-hip ratio). The levels are inversely related to BMI and adipose tissue mass, both of which are downregulated in overweight and obese individuals. Other epidemiologic researchers have found a strong negative relationship between adiponectin and BC, even after adjusting for adiposity [13].

According to Jiralerspong et al.'s study, altered biology leading to more aggressive tumors in obese people is a substantial component of this inferior prognosis. Higher BMI is related to poorer outcomes in the ER-positive subset but not in the ER-negative and HER2-positive subsets, according to ER and HER2 marker status analysis. The association between diabetes and variations in recurrence-free survival (RFS) and overall survival (OS), but not breast cancer-specific survival (BCSS), shows that diabetic individuals have more BC recurrences and overall death than nondiabetic patients, but not death from BC itself. This phenomenon might be due to differences in diagnosis, concomitant diseases, and therapy. This study further speculates that diabetic patients may have underlying biology that favors tumor recurrence [16].

IR was shown to be interrelated with Ki-67 expression in Nam et al.'s research. Ki-67 is a nuclear antigen present in proliferating cells that may be used as a marker for cell proliferation. According to the Ki-67 expression status and BC subtype, the mean insulin and HOMA-IR values appeared considerably varied, and the serum levels were unaffected by histologic grade, ER status, or HER2 status. This group of patients had IR-large tumors and high Ki-67 levels, indicating that IR might be a valuable prognostic marker. However, further independent confirmation of IR's use as a prognostic marker is required. The aromatization of androstenedione leading to a hormonal shift might play a role in the rise in visceral adiposity attributed to IR in postmenopausal women seen in this study. Adipocyte-secreted substances have been shown to promote BC directly by inducing anti-apoptotic transcriptional programs and proto-oncogene stability. These findings might help identify subgroups that could benefit from specialized therapies to overcome IR [6].

Lastly, metformin's direct antitumor impact in nondiabetic postmenopausal women with ER-positive BC is now being studied in prospective and randomized controlled studies. Such clinical studies' findings are expected to increase existing knowledge shortly significantly [6].

Another recent study noted that even a single component of MetS, such as high waist circumference, blood pressure, fasting glucose, or triglycerides, was strongly associated with an increased risk of BC mortality, regardless of other well-known prognostic factors like age, tumor stage, immunohistochemical (IHC) subtypes, or therapy. It is also to be noted that patients with MetS were shown to be more likely to be older and postmenopausal than those without the disease. At a median follow-up period of 7.1 years, they discovered that MetS was substantially related to an increased risk for death in general and from BC in early BC patients undergoing neo-adjuvant treatment, and the existence of MetS at the time of diagnosis had no bearing on the therapy chosen [11].

**Figure 3:**
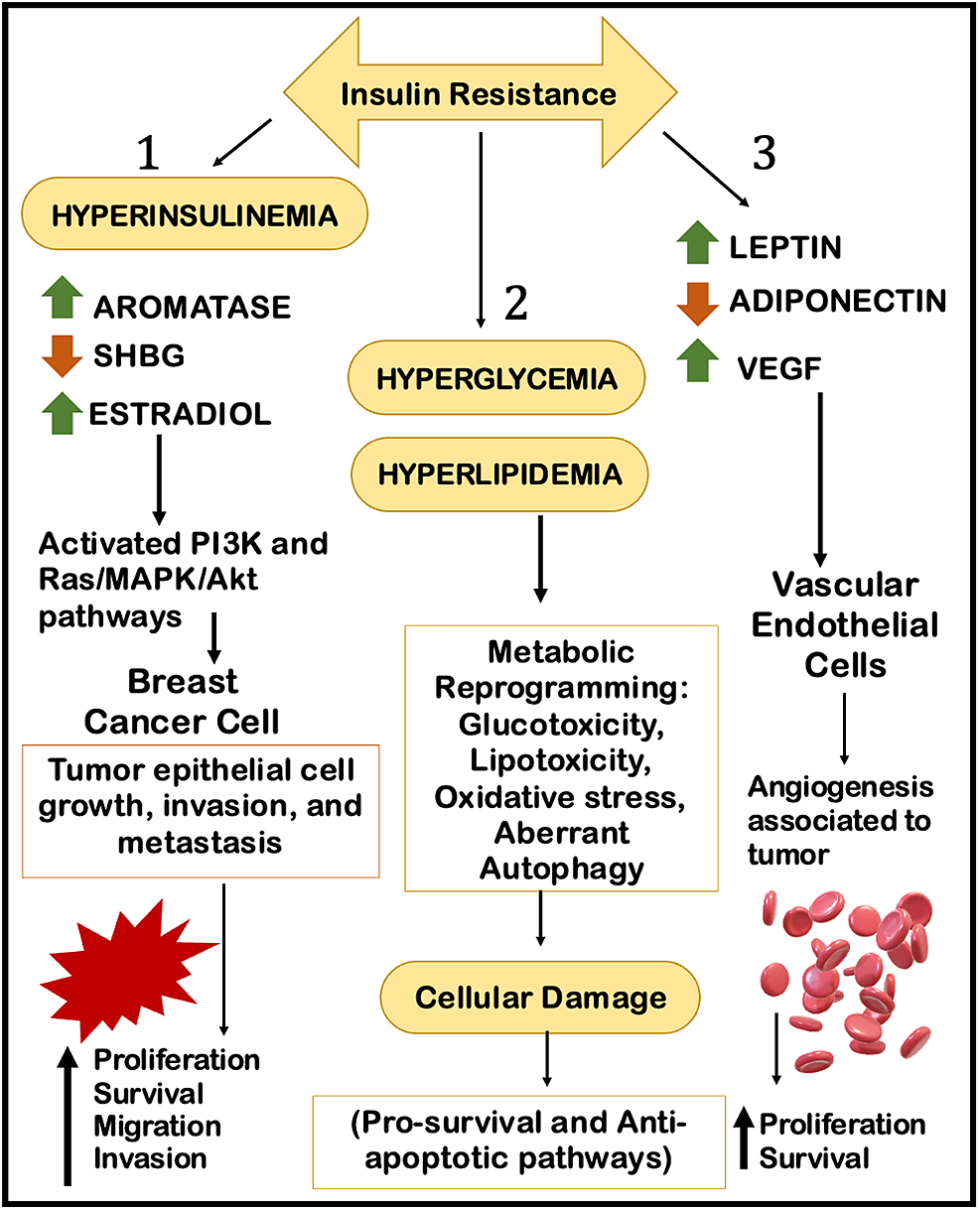
The molecular mechanism of insulin resistance and breast cancer Figure created by the authors on Microsoft PowerPoint (Microsoft Corporation, Redmond, WA). SHBG: sex hormone-binding globulin; VEGF: vascular endothelial growth factor; PI3K: phosphoinositide 3-kinase; Ras: rat sarcoma; MAPK/Akt: mitogen-activated protein kinase/protein kinase B.

Glucose and IR have been shown to increase the proliferation of cancerous cells. Insulin stimulates the synthesis of IGF-1 in malignant cells, resulting in hyperactivation of the Ras/MAPK and phosphoinositide 3-kinase/protein kinase B (PI3K/Akt) pathways and an increase in serum-free estrogen levels by lowering the concentration of SHBG. Obesity causes a low-grade chronic inflammation as well as promotes estrogen synthesis, as the aromatase enzyme synthesizes estrogens in adipose tissue from circulating androgens. Reduced levels of anti-inflammatory cytokines (such as adiponectin) and increased levels of proinflammatory cytokines (such as tumor necrosis factor α (TNFα), interleukin (IL)-1β, IL-6, and IL-8), which can have mitogenic, anti-apoptotic, and angiogenic effects, accelerate tumor progression. Significantly, interactions with BC cells may stimulate the transition of mammary adipocytes into "cancer-associated adipocytes" (CAA), which may promote tumor development and progression through lipolytic activity and adipokine release [11]. Figure 3 depicts an overview of the molecular mechanism of IR and BC [20,21].

Limitations

This study has a few limitations. The study only focuses on observational studies and individual case reports or case series, and randomized trials were not analyzed to maintain uniformity of the data studied. A particular geographical location was not focused to view a worldwide aspect of breast neoplasm, where cultural/conservative approaches including alternative therapies could influence the reporting for this malignancy. The study sample in this review ranged from a moderate number to a larger population, and the female gender was only explored due to the increasing trend seen for both IR and BC.

## Conclusions

MetS are simply characterized as IR in which hyperinsulinemia and adipokine dysregulation are interconnected to either overweight or obesity, both of which indirectly contribute to a poor prognosis of BC. According to the recent studies on the subject, obesity and MetS have both been related to an increased risk of BC, especially in postmenopausal women; nevertheless, their relative contributions remain incompletely understood. Despite this restriction, it is speculated that the primary mechanism of BC in such individuals could be elevated estrogen production by adipose tissue and obesity, leading to physiologically aggressive BC irrespective of menstrual status or tumor estrogen dependence. Moreover, T2DM and concomitant obesity are associated with BC risk and prognosis, where insulin directly boosts cell proliferation. It is also possible that persistent adipose tissue inflammation, rather than BMI-defined obesity, is linked to an elevated risk of postmenopausal estrogen-dependent BC in IR women. In addition to the known relationship between general obesity and the risk of BC, central obesity and circulating fasting insulin and glucose have been connected to the development of this prevalent malignancy. Hence, it is to be understood that IR could not only increase the risk but also alter the prognosis of BC. For this purpose, one should be aware of such an association at a primary level itself and provide recommendations, whether being lifestyle modifications and/or corrections of IR, while also providing regular screening services not only in the developed country but also in developing and low-income countries as well.
